# Characterisation of the genomic architecture of human chromosome 17q and evaluation of different methods for haplotype block definition

**DOI:** 10.1186/1471-2156-6-21

**Published:** 2005-04-25

**Authors:** Eleftheria Zeggini, Anne Barton, Stephen Eyre, Daniel Ward, William Ollier, Jane Worthington, Sally John

**Affiliations:** 1 Centre for Integrated Genomic Medical Research, University of Manchester, Manchester, UK; 2 Wellcome Trust Centre for Human Genetics, University of Oxford, Oxford, UK; 3 arc Epidemiology Unit, University of Manchester, Manchester, UK

## Abstract

**Background:**

The selection of markers in association studies can be informed through the use of haplotype blocks. Recent reports have determined the genomic architecture of chromosomal segments through different haplotype block definitions based on linkage disequilibrium (LD) measures or haplotype diversity criteria. The relative applicability of distinct block definitions to association studies, however, remains unclear. We compared different block definitions in 6.1 Mb of chromosome 17q in 189 unrelated healthy individuals. Using 137 single nucleotide polymorphisms (SNPs), at a median spacing of 15.5 kb, we constructed haplotype block maps using published methods and additional methods we have developed. Haplotype tagging SNPs (htSNPs) were identified for each map.

**Results:**

Blocks were found to be shorter and coverage of the region limited with methods based on LD measures, compared to the method based on haplotype diversity. Although the distribution of blocks was highly variable, the number of SNPs that needed to be typed in order to capture the maximum number of haplotypes was consistent.

**Conclusion:**

For the marker spacing used in this study, choice of block definition is not important when used as an initial screen of the region to identify htSNPs. However, choice of block definition has consequences for the downstream interpretation of association study results.

## Background

Recent advances in high-throughput genotyping technologies have realised the possibility of performing large-scale, high-resolution genetic studies in human complex diseases. Single nucleotide polymorphisms (SNPs) have become the markers of choice due to their frequent occurrence, simple mutational dynamics and the fact that they lend themselves to automated allele calling [1,2]. The number of SNPs with allele frequencies higher than 10% has been estimated to exceed 5,000,000 [3]. Exhaustive genome-wide association studies, thereby, reach prohibitive costs and require ultra-high throughput technologies. Comprehensive SNP screening of regions or genes of interest is both inefficient and unnecessary, as information redundancy can arise from linkage disequilibrium (LD). A common strategy in complex disease association studies is the selection and genotyping of a subset of SNPs, assumed to be in LD with the untested polymorphisms. In the past, association study designs have not selected markers on a strong scientific basis, due to restricted comprehension of LD patterns. Gaining a better understanding of the LD blueprint of the human genome can now facilitate disease gene mapping, as sets of non-redundant SNPs can be employed to design cost-effective strategies [4-6]. SNP maps utilised by current genetic studies concentrating on chromosomal regions cover a wide spectrum of marker spacing intervals, ranging from ~50 kb [7,8] to 15–20 kb [9,10] to high resolution maps of approximately 1 SNP every kb [11,12].

Patterns of LD across the genome have been shown to be variable and found to be a property of individual chromosomal regions rather than a simple monotonic function of physical distance between markers [6,9,13-15]. Regions of low haplotype diversity interspersed by regions of low LD (termed haplotype blocks) have been empirically identified and proposed to constitute a ubiquitous feature of the genome [16-18]. Their presence has triggered funding of the Haplotype Map project, leading to the generation of a genome-wide index of common blocks. Characterisation of haplotype blocks can provide association studies with a shortcut to screening chromosomal regions for the presence of disease variants through the identification of haplotype-tagging SNPs (htSNPs) and can additionally aid in interpreting the results of initial scans through knowledge of the underlying genetic architecture [5,19-22].

The potential benefits of utilising haplotype blocks may, however, be challenged by concerns regarding their consistency and, hence, applicability to different populations, the information loss incurred by examining common variation and the arbitrary choice of block definition [23]. Different studies investigating the structure of haplotype blocks have used distinct definitions based on various subjective criteria. Block definition methods can be broadly classified into three categories: those based on measures of LD [16,24], those based on haplotype diversity [11,25,26] and those combining both approaches [9,27]. Methods based on LD measures generally define blocks as regions in which all pairwise LD coefficients exceed a subjective threshold. Methods based on haplotype diversity generally define blocks as regions in which a small, arbitrary number of haplotypes accounts for a predefined percentage of the observed variation. The consensus finding is that denser marker maps, larger sample sizes and use of common variants lead to shorter blocks [23,24]. However, the extent of difference in block structure, to which distinct haplotype block definitions and thresholds may result in, remains unclear. The size and number of generated blocks could have an impact on the downstream analysis of association studies and could, therefore, influence the design of fine mapping strategies to identify disease-causing variants.

In the present study, we address these issues by applying different haplotype block definition criteria to 137 SNPs, in order to describe the genetic architecture of a 6.1 Mb region of 17q in a set of 189 unrelated healthy individuals. Employing methods based on both LD measures and haplotype diversity, we evaluate their relative merits and limitations, given our median marker spacing of 15.5 kb. Comparing the generated underlying block structures, we assess the usefulness and applicability of distinct methods in genetic association studies of complex human diseases.

## Methods

### Subjects

DNA from a cohort of 189 healthy, unrelated, UK individuals of Euroepan ancestry was studied. Individuals were recruited from general practice or were blood donors. The collection was approved by the regional ethics committee.

### Markers and genotyping

One hundred and thirty seven SNPs dispersed over 6.1 Mb of the human chromosomal 17q region were examined. We are currently investigating these markers as part of a fine mapping study for the identification of rheumatoid arthritis susceptibility genes. SNPs were selected from the SNP Consortium database [28] to span the region in equally spaced intervals. The SNP map of successfully genotyped markers was constructed based on the November 2002 Freeze of the Human Genome Sequencing Project, available through the UCSC Genome Browser [29].

Methodological details are available upon request from the authors and SNP IDs can be found in Additional file 1 [see Additional file 1]. Briefly, SNPs were genotyped using either the primer extension SNaPshot™ method (Applied Biosystems, CA, USA) through use of an ABI Prism 3100 DNA Analyzer and GeneScan^® ^analysis software (Applied Biosystems, CA, USA), or the allelic discrimination 5' nuclease assay (TaqMan^®^, Applied Biosystems, CA, USA) through use of an ABI Prism 7700 platform (Applied Biosystems, CA, USA). All SNP genotype calls were independently checked by two individuals.

### Haplotyping

Departure from Hardy Weinberg equilibrium was initially assessed for each SNP. None of the SNPs were found to deviate from Hardy-Weinberg equilibrium significantly. Haplotypes were then inferred using the expectation-maximisation (EM) algorithm, either through the HelixTree™ (Golden Helix, Inc, Montana, USA) or the snphap (David Clayton, Cambridge, UK) software packages. Convergence of the algorithm was checked by repeating the haplotype estimation process 3 times, ensuring that identical results were generated.

### Pairwise LD

Using SNP genotypes, the pairwise LD measure of D' was calculated. As values of D' can be overestimated with rare allele frequencies [20], the LD correlation coefficient r^2 ^was additionally calculated for all pairs of SNPs. Observed D' and r^2 ^values were sorted according to distance between the corresponding marker pairs. Running average D' and r^2 ^values for sliding windows of 2 consecutive observations were estimated and plotted.

### Haplotype block definitions

Haplotype block definitions were applied to the total set of 137 SNPs, as well as to the set of SNPs with allele frequencies exceeding 0.2 separately, in order to assess the effects of variant frequency on block structure.

Definition 1: The block definition method based on the D' measure of LD, employed by Gabriel et al. 2002 [16], was applied to the SNP genotype data through the HaploView software package (MJ Daly and JC Barrett, Whitehead Institute, MA, USA). Briefly, a block was defined as a region in which less than 5% of SNP pairs had a D' upper confidence bound less than 0.9. In addition, blocks consisting of 2 SNPs could span up to 20 kb and blocks of 3 or 4 SNPs could span up to 30 kb. Blocks were not allowed to overlap.

Definition 2: a; A simplified block definition method, also based on LD measures, was used. A haplotype block was defined as a region in which over 95% of all pairwise r^2 ^LD correlation values exceeded 0.4. The same block length constraints as in Definition 1 were imposed, but a less rigid threshold was employed for stringency evaluation purposes. Blocks were allowed to overlap.b; For the subset of common SNPs, an additional method, based on D' values that tend to be overestimated for rare allele frequencies, was also employed. A haplotype block was defined as a region in which over 95% of all pairwise D' values exceeded 0.4. Blocks were allowed to overlap.

Definition 3: The block definition method proposed by Wang et al. 2002 [24] was applied to the dataset. Briefly, a block was defined as a region in which, for all possible pairs of markers, less than four gametes were observed (D' = 1). Blocks were allowed to overlap.

Definition 4: A block definition method based on haplotype diversity was developed. For a set of n SNPs, the maximum number of haplotypes observed in the absence of recurrent mutation and / or recombination is n+1. Therefore, a haplotype block was defined as a region consisting of n SNPs, in which n+1 haplotypes could account for at least 95% of the observed variation. Taking each SNP as a seed, blocks were expanded or contracted to find the optimal window. Haplotype blocks were allowed to overlap.

Definition 5: A further block definition method based on LD measures, as applied in the HaploView software package (MJ Daly and JC Barrett, Whitehead Institute, MA, USA), was employed. A haplotype block was defined as a region in which all of the pairwise D' values exceeded 0.8. Blocks were not allowed to overlap.

### htSNP identification

The minimum number of SNPs that capture the maximum number of haplotypes (htSNPs) [30] were determined for each resulting block of each definition method. The htSNP2 programme (David Clayton, Cambridge, UK) implemented in Stata and the HaploView software package (MJ Daly and JC Barrett, Whitehead Institute, MA, USA), both making use of the EM algorithm, were employed to identify htSNPs. The r^2 ^correlation measure, calculating the ability to predict frequencies at a series of loci using just the subset of htSNPs, was set to the stringent threshold of 0.95 for the htSNP2 programme. Good correspondence was observed between the two methods. HtSNPs were additionally identified with a set htSNP2 r^2 ^threshold of 0.80, in order to check consistency under varying degrees of stringency.

## Results

**Figure 1:**
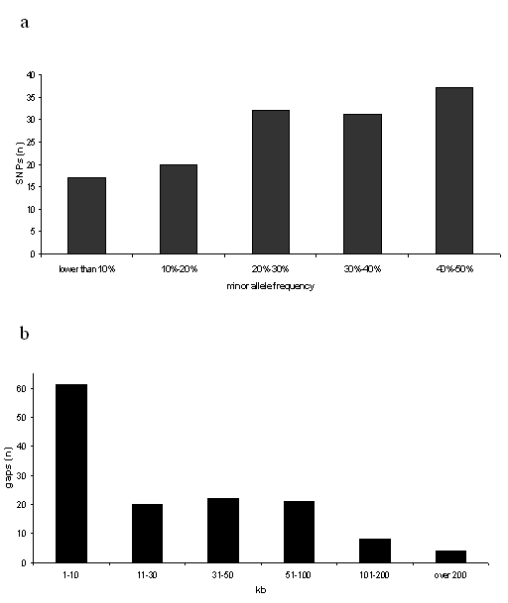
a; The distribution of minor allele frequencies for the 137 SNPs used in this study. The bias toward common alleles is inherent to the sampling of markers from publicly available databases. b; The distribution of physical gaps between the 137 SNPs used in this study (median spacing 15.5 kb).

**Figure 2:**
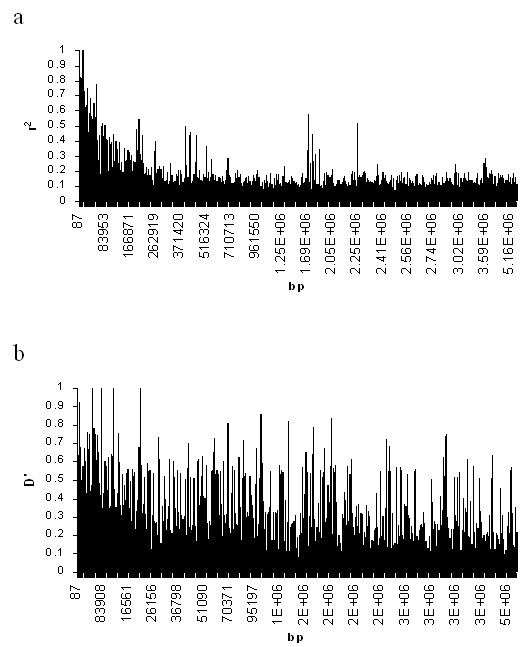
Running average values of LD measures for sliding windows of 2 SNPs for the 137 markers studied. a; Variability of r^2^. Patterns of decay of LD in this dataset correlate well with observations in different regions of the human genome. b; Variability of D'.

Minor allele frequencies of the 137 SNPs studied ranged from 0.06 to 0.5 (Figure 1a), with an average frequency of 0.29. The observation that 100 (73%) of the SNPs were common (frequencies greater than 0.2) could be explained by ascertainment bias, as all SNPs were selected from publicly available databases [31]. Inter-SNP distances ranged from 55 bp to 951 kb (median spacing 15.5 kb). The marker map contained 4 gaps longer than 200 kb (Figure 1b). Plotting the moving average of r^2 ^exhibited an overall negative correlation between LD and physical distance, with some variability observed for distant SNPs exhibiting evidence for association (Figure 2a). The moving average of D' demonstrated extreme variability in the distribution of LD and its decay with distance, an artefact stemming from low allele frequencies (Figure 2b).

**Figure 3:**
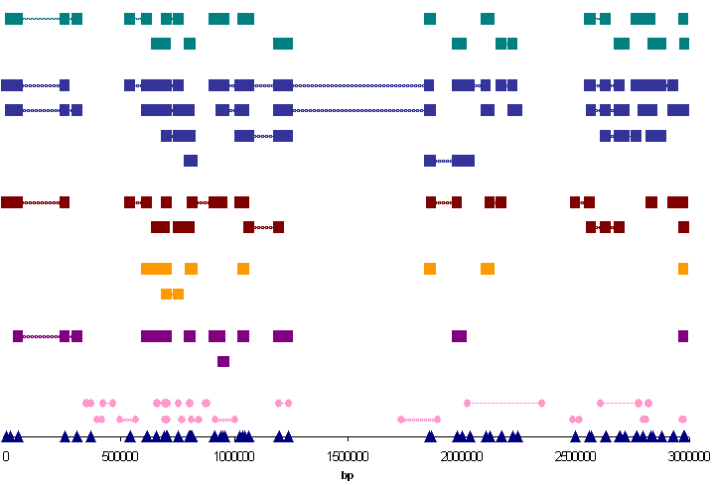
Snapshot of haplotype block organisation on 17q. Blocks identified by each of the 5 Definitions for the first 3 Mb of the region are depicted. SNPs are shown as triangles according to their relative spacing. Genes in the region are shown in pink (circles denote the start and end points of genes). Haplotype blocks are colour-coded according to the Definition used to characterise them: Definition 1 [16] is in purple; Definition 2 (modification of [16]) is in orange; Definition 3 [24] is in red; Definition 4 (n+1 method) is in blue; Definition 5 (D' high threshold method) is in green. Squares represent the SNPs that fall within the defined blocks and lines extend across each haplotype block. Adjacent and overlapping blocks are depicted in consecutive rows.

To characterise and compare block patterns, 5 distinct haplotype block definitions were applied to the SNP genotype data. The same sets of parameters reflecting on underlying block structure were determined for each method (Table 1). Therefore, in order to gain an understanding of how each definition portrayed the region's genetic architecture, the number of resulting haplotype blocks, the average length and SNP content of blocks, as well as the proportion of sequence and markers covered by blocks were evaluated. The process was repeated for the subset of common SNPs only (Table 2). In general, methods based on LD measures (Definitions 1, 2, 3 and 5) resulted in fewer, shorter blocks, while the haplotype diversity-based method (Definition 4) provided an overall greater coverage of the region (Figure 3). Although map density was sparser in the group of common SNPs (median spacing of 1 SNP / 29.5 kb), the inclusion of markers with minor allele frequencies less than 0.2 appeared to have an overwhelming effect for the majority of definitions and generally resulted in reduced coverage of the sequence examined.

**Table 1:** Haplotype block characteristics according to different definition methods, applied to the total group of 137 SNPs.

Definition^a^	n blocks	Average block length (kb)	Average n SNPs/block	% of sequence covered	n SNPs in blocks (%)
Definition 1	20	28.3	2.6	9.3	52 (38)
Definition 2	19	16.5	2.6	5	46 (33.6)
Definition 3	32	24.2	2.1	12.7	62 (45.3)
Definition 4	60	130.7	4.3	85.8	130 (95)
Definition 5	38	42.0	2.9	26.2	111 (81)

^a^Definition 1 [16], Definition 2 (modification of [16]), Definition 3 [24] and Definition 5 (D' high threshold method) were based on measures of LD, whereas Definition 4 (n+1 method) was based on haplotype diversity.

**Table 2:** Haplotype block characteristics according to different definition methods, applied to the subset of 100 common SNPs (minor allele frequency >0.2).

Definition^a^	n blocks	Average block length (kb)	Average n SNPs/block	% of sequence covered	n SNPs in blocks
Definition 1	17	31.1	2.6	8.7	44
Definition 2a	14	10.9	2.6	2.4	32
Definition 2b	21	10.8	2.5	3.3	53
Definition 3	19	18.4	2	5.7	33
Definition 4	39	107.0	3.8	55.4	85
Definition 5	27	41.1	2.7	18.2	73

^a^Definition 1 [16], Definitions 2a and 2b (modifications of [16]), Definition 3 [24] and Definition 5 (D' high threshold method) were based on measures of LD, whereas Definition 4 (n+1 method) was based on haplotype diversity.

**Figure 4:**
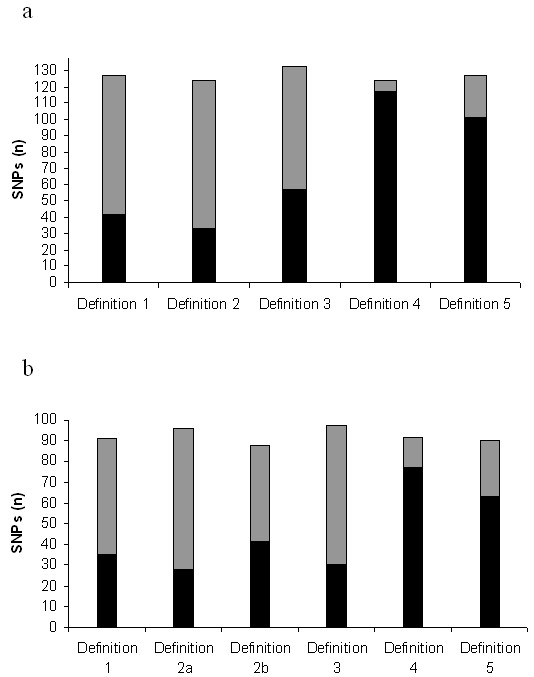
Number of SNPs that need to be genotyped, in order to capture the majority of variation in the region, according to the different haplotype block definition methods. a; In the total group of 137 SNPs. b; In the subset of 100 common SNPs (minor allele frequency >0.2). The number of htSNPs falling within haplotype blocks is denoted by black, while the number of SNPs that need to be typed but are not included in blocks, is depicted in grey.

Figure 4 depicts the total number of markers that were necessary to capture most variation in this chromosomal region, as derived from each definition, for the total group of SNPs and for the subset of common SNPs. To calculate this parameter, the number of htSNPs identified for each haplotype block was added to the number of SNPs that were not encompassed within blocks. Genotyping of a similar proportion of markers appeared to be necessary across the different definitions for all markers (90.5% to 96.4%) and for the common SNPs (88% to 97%) at the stringent r^2 ^correlation measure threshold of 0.95. The observation that the vast majority of SNPs needed to be typed in order to capture most of the chromosomal variation was confirmed when the r^2 ^threshold was decreased to 0.80.

## Discussion

Recently, numerous groups have studied the presence and distribution of haplotype blocks in the human genome, each proposing and utilising distinct block definition methods. Each study has examined different numbers of SNPs, dispersed throughout differently sized chromosomal regions, at varying minor allele frequency and map spacing, making use of diverse sample sizes [9,11,16,25,27]. The underlying design of this study reflects a realistic scenario, in which a region of several Mbs has been implicated in susceptibility to a human complex disease and is being refined through LD mapping. The ascertainment of SNPs through publicly available databases additionally represents practically favoured selection processes, giving rise to a well-recognised bias toward common polymorphisms and leading to, in this case, a median marker spacing of 15.5 kb (equivalent to that used by Dawson et al. 2002 [9]). This SNP density would be expected to give rise to apparently longer haplotype blocks compared to denser maps, such as the HapMap. The selection of unrelated individuals is in keeping with the current trend toward population-based, rather than family-based, studies and the pragmatic sample size of 378 chromosomes allows effective *in silico *haplotype inference.

The extent and variability of inter-marker LD in this study corroborates recent findings of distribution irregularity [6,9,13-15]. The observation that LD does not decay uniformly with physical distance exemplifies the need for haplotype block structure determination. Evidence for disequilibrium has been detected between SNPs as far as 1.7 Mb apart, extending much further than previously indicated through simulation [32]. The discrepant LD patterns derived from distinct LD measures highlight the need for caution in interpreting and comparing studies, especially when using D', for which there is an upward bias with small sample sizes and rare allele frequencies.

Haplotype block definition methods employed in this study have been based both on measures of LD and on haplotype diversity, and applied to the same dataset, thus enabling direct comparison of their performance. Definitions 1 [16] and 3 [24] have been proposed in recent studies of block structure in human genomic regions. Definition 2, based on measures of LD, was developed as a simplified modification of Definition 1 [16] to accommodate less stringent thresholds and criteria. Definition 5 was used to reflect block structure based on criteria setting a high threshold of D', but no length constraints. In addition, a novel diversity-based method was developed (Definition 4), which does not impose strict block boundaries and incorporates the notion of recombination events and recurrent mutation, factors known to diminish inter-marker LD.

All methods provided evidence for a block-like organisation of the genetic variation in the chromosomal region under investigation on 17q, characterised by marked differences among the distinct definitions, in accordance with previous observations [33,34]. Overall, the haplotype diversity-based method (Definition 4) gave a more comprehensive coverage of the sequence, resulting in a higher number of blocks with a longer average physical size, compared to definitions based on measures of LD. These findings are in agreement with a recent study, comparing the performance of one LD-based with one haplotype-based block definition [34]. Such differences in characterising the underlying genetic architecture of a region could have implications in the interpretation of association studies and the design of subsequent strategies. Inclusion of a greater proportion of the region into blocks maximises the chances that a significant association observed through a first scan will be encompassed within a haplotype block, thus delineating the interval on which further fine mapping attempts can be focused. Localisation of a positive result outside the boundaries of defined blocks would necessitate more intensive genotyping efforts targeted to the surrounding region. Although extended coverage of a sequence interval may prove useful, it could be artificial, stemming from methodological inadequacies, thereby leading to a false representation of the underlying genomic structure. Although the newly developed Definition 4 (n+1 method) resulted in higher sequence coverage, the lack of any LD constraints in this definition could lead to a falsely inflated detection of short haplotype blocks in cases of SNPs with rare minor allele frequencies. Of the LD-based methods, Definition 5 (D' high threshold method) provided the highest coverage of the sequence studied, although approximately 74% of the region did not fall into blocks. The observed inconsistency among methods illustrates the subjectivity of haplotype block definition and prevents the conclusive characterisation of the region's block structure.

Haplotype block assignment was found to change not only due to inter-method differences, but also as a result of altering parameters within the same set of definition criteria. These observations corroborate previous findings [34]. Application of the same methods to the subset of common SNPs led to an overall reduction in the proportion of the sequence covered. Although marker spacing was sparser and the number of polymorphisms examined smaller, high minor allele frequency had an overwhelming effect on haplotype block size, generally resulting in shorter blocks. Definition 1 [16], however, appeared to be robust to such changes, therefore offering a possible mechanism to achieve consistency in block structure between SNP subgroups of varying allele frequencies. Among LD-based definitions, use of D' rather than r^2 ^resulted in the generation of more haplotype blocks and in an increased coverage of the region. Similarly to when applied to the total set of markers, the newly introduced Definition 4 (n+1 method) produced the highest sequence coverage when examined in the subset of common SNPs only, although a proportion of relatively infrequent SNP pairs in low LD could have been falsely categorised as blocks.

Comparison of different haplotype block definitions in characterising the genomic organisation of the human chromosomal region 17q revealed discrepancies among methods and could, therefore, raise concerns about both the suitability of ad hoc approaches for the crude identification of block structure, as well as the validity of the notion of haplotype blocks as a genomic feature. However, the observed overlap in SNPs encompassed within blocks across all definitions used, indicated an underlying genetic architecture captured by all methods. Concordance among all definitions was additionally exhibited in calculating the subset of SNPs necessary to encapsulate the vast majority of genetic variation in the region. Selection of block definition method appeared to be irrelevant when genotyping a sample subset for all markers in order to identify haplotype tagging SNPs. In this study, the percentage of markers that needed to be typed was extremely high (over 90%), indicating that the marker map density employed was not suited to achieving significant cost-effectiveness through htSNP characterisation. Reassuringly, as all methods suggested typing the same number of markers, they probably also carry equal chances of detecting a possible association due to LD. The differences, however, would arise in interpreting downstream results and developing follow-up strategies.

The proposal of taking advantage of haplotype blocks to inform strategic designs in genetic association studies constitutes a welcome step forward, rather than a panacea, for the field of human complex disease genetics. In a realistic study design, the choice of block definition method could be of consequence in designing and interpreting genetic association scans. In addition, the inclusion of SNPs with rare minor allele frequencies appears to convolute, rather than clarify, the underlying genomic structure. Given the marker density of 15.5 kb, a whole genome scan by association would require approximately 100,000 SNPs to be genotyped. The findings of this study indicate that such a spacing would not be adequate for characterising the genomic architecture in sufficient detail through a haplotype block definition approach. Further issues inherent to the characterisation and utilisation of chromosomal underlying block structure need to be addressed in both real and simulated datasets, in order to clarify the settings in which haplotype blocks may prove useful.

## Authors' contributions

EZ participated in study design, carried out the statistical analyses and drafted the manuscript. AB participated in study design and coordinated genotyping efforts. SE and DW carried out the SNP genotyping. JW and WO participated in study design. SJ participated in study design and coordination and helped draft the manuscript. All authors read and approved the final manuscript.

## Supplementary Material

Additional File 1 SNP IDs and chromosomal locations at the time of SNP selection. Short description of the data: Additional file 1 contains a full list of SNPs used for the analyses. The SNPs are identified by their rs number. Where rs numbers are not available, the ABI assay-on-demand ID has instead been included. Click here for file
